# Physician Documentation of Access to Firearms in Suicidal Patients in the Emergency Department

**DOI:** 10.5811/westjem.2019.7.42678

**Published:** 2019-08-20

**Authors:** Sonya Naganathan, Kristen L. Mueller

**Affiliations:** Washington University in St. Louis School of Medicine, Department of Emergency Medicine, St. Louis, Missouri

## Abstract

**Introduction:**

Suicide is the 10th leading cause of death in the United States. An estimated 50% of these deaths are due to firearms. Suicidal ideation (SI) is a common complaint presenting to the emergency department (ED). Despite these facts, provider documentation on access to lethal means is lacking. Our primary aim was to quantify documentation of access to firearms in patients presenting to the ED with a chief complaint of SI.

**Methods:**

This was a cross-sectional study of consecutive patients, nearly all of whom presented to an academic, urban ED with SI during July 2014. We collected data from all provider documentation in the electronic health record. Primary outcome assessed was whether the emergency physician (EP) team documented access to firearms. Secondary outcomes included demographic information, preexisting psychiatric diagnoses, and disposition.

**Results:**

We reviewed 100 patient charts. The median age of patients was 38 years. The majority of patients had a psychiatric condition. EPs documented access to firearms in only 3% of patient charts.

**Conclusion:**

EPs do not adequately document access to firearms in patients with SI. There is a clear need for educational initiatives regarding risk-factor assessment and counseling against lethal means in this patient cohort.

## INTRODUCTION

Firearm-related injury and death is a significant and expensive public health issue. Recent data from the Centers for Disease Control and Prevention (CDC) report suicide as the 10th leading cause of death in the United States, with firearms reported as the cause of death in an estimated 50% of these cases.^1^ Additionally, the direct medical costs related to firearm injuries nationally are as high as $2.9 billion dollars per year.^2^

Firearm injuries are also a common reason patients present to the emergency department (ED). ED visits for firearm injuries occurred at an estimated national incidence of 25.3 ED visits per 100,000 people between 2006 and 2014; the burden of non-fatal firearm injuries is likely underestimated at 2.4 times that of fatal injuries.^2^ Given the magnitude of firearm-related injury and death in the U.S., there is a clear public health need for educational initiatives and patient-centered interventions regarding firearm safety.

Additionally, the ED is a frequent point of access to care for patients with suicidal ideation (SI).

Previous work demonstrated that many patients have their first point of contact with the mental healthcare system less than one month before suicide is attempted, and that disadvantaged groups are less likely to have access to outpatient mental healthcare.^3^ For this reason, many people present to EDs in times of suicidal crisis. In 2013 the Agency for Healthcare Research and Quality found that 903,400 ED visits were related to SI; this constitutes an estimated 12% average annual increase in the population-based rate of ED visits for SI since 2006.^4^ Survey data from 9708 individuals suggested 64% of patients with SI and 79% of patients with suicide attempt sought general medical or subspecialty care in the year prior to presentation.^5^ However, only 44% of those patients with SI sought mental health treatment during that time.^5^

In evaluation of healthcare providers’ perception of responsibility when it comes to the assessment of access to firearms, a Betz et al. survey of ED providers revealed that only 43% believed that “‘most’ or ‘all’ suicides are preventable.”^6^ Another study noted that 57% of ED nurses and physicians believed it was the responsibility of the ED nurse to ask about access to firearms, and 71% felt that it was the emergency physician’s (EP).^7^ However, in this study,84% of respondents felt that it was the responsibility of the psychiatrist to ask patients about their access to firearms.

Given these beliefs, some skepticism exists about the EP’s role in risk assessment of lethal-means access in suicidal patients. In subsequent work, Betz et al. found low rates of EP documentation of access to lethal means in suicidal patients.^8^ However, no clear documentation guidelines exist for emergency providers on risk assessment in such situations. Without clear documentation in the electronic health record (EHR), it is difficult to know whether emergency providers are asking these patients about access to firearms.

### Objectives

The goal of this study was to quantify provider documentation of access to firearms in patients who present to the ED with a chief complaint of SI. We hypothesized that EPs did not consistently document access to firearms in patients presenting with SI. Our secondary outcomes were to assess demographic information, preexisting psychiatric diagnoses, and disposition in this cohort.

## METHODS

### Study Design and Setting

This was a cross-sectional study of consecutive patients presenting to the ED with SI. The study sites included two EDs; the first is an urban, academic, tertiary-care referral center with approximately 95,000 ED visits per year, and the second is an affiliated community hospital with approximately 11,500 ED visits per year. Patients presenting to these EDs with SI are cared for by attending EPs and resident physicians in emergency medicine and psychiatry. This observational study is reported in accordance with the Strengthening the Reporting of Observational Studies in Epidemiology (STROBE) Statement: Guidelines for Reporting Observational Studies.^9^ This study was approved by the institutional review board (IRB).

### Selection Criteria

We reviewed the medical records of consecutive patients who presented to an ED between July 1, 2014, and July 31, 2014, with a nursing triage chief complaint of SI. This brief study period was used as a needs assessment in the development of a subsequent, prospective, ED-based quality improvement program on counseling on access to lethal means. Patients with multiple ED presentations during the study period were included once at the time of their index visit.

### Variables

We collected data on the following variables: age; sex (male, female); race (African American, Caucasian, other); marital status (married, single, romantic partner, unknown); psychiatric diagnosis (anxiety, bipolar, depression, psychosis not otherwise specified, schizophrenia, other, none); disposition (admit, discharge, transfer); and whether there was EP documentation of patient access to firearms in this cohort.

### Data Sources

We identified patient encounters through query of the EHR for ED patients presenting with a chief complaint of SI. These data were stored on a password-protected secure server in compliance with our institution’s IRB. Data were queried by S.N. from the entire ED chart including EP documentation, nursing documentation, psychiatry consult notes, and pre-hospital documentation in the EHR.

### Analysis

We presented the data as descriptive statistics. Categorical data is presented in both raw count and percentages.

## RESULTS

Of the total 100 patient encounters included in our study, 99 of these patients presented to the academic center, and one presented to the community ED. We excluded eight patient encounters for repeat visits during the study period, and one was excluded for a complaint that was not related to SI or mental health. Patient characteristics are described in the Table. The median age was 38 years. Of those patients presenting to the ED for SI, 64% were male and 53% were Caucasian; 54% were single, and 13% were married. The majority of patients had an underlying psychiatric diagnosis. Nearly equal thirds of patients were admitted, transferred to other psychiatric hospitals for admission, and discharged.

We found that EPs documented access to firearms in only 3% of the study population; 97% of these patient encounters did not contain documentation of access to firearms (Figure). Of the 100 patients queried, psychiatry was consulted on 81 patients;78% of these patients had access to firearms assessment documented by the psychiatry consultant.

## DISCUSSION

We found that EPs did not routinely document access to firearms in suicidal patients. Of particular concern, this discussion of access to firearms—the most common method of completed suicide in the U.S.—was not documented by EPs in 97% of patients. These findings support previous work that indicate physicians in general, and EPs specifically, are not asking suicidal patients about their access to firearms.^8^ Although the psychiatry consultants had improved documentation rates of patient firearm access in this cohort, they too had a significant gap, with firearm access documentation absent in 22% of psychiatry consult notes. Ultimately, EPs are responsible for all aspects of ED care and it is important for EPs, specifically, to document this risk factor.

While the sample size and time period for this study were small, the marked lack of EP documentation on this issue was the wake-up call needed to launch a prospective, ED-based quality improvement program on counseling on access to lethal means in patients presenting with SI. As firearms are the most common way that Americans die by suicide, we submit these data for consideration with the hope that similar brief analyses at other institutions will improve discussion, documentation, and ED-based counseling on access to, and safe storage of, firearms and other lethal means in ED patients at times of suicidal crisis.

## LIMITATIONS

This study was limited by its retrospective design and small sample size, and no statistical software was required for the analysis. Of note, primary endpoints for this study were limited by provider documentation in the EHR; it is possible that more physicians had discussed access to lethal means with their patients and did not document these conversations.

## CONCLUSION

The ED is uniquely positioned to evaluate for and disseminate information regarding access to and safe storage of firearms at times of suicidal crisis. As lethal means counseling is a core practice guideline in suicide risk assessment and management, the Suicide Prevention Resource Center designed an online training module, Counseling on Access to Lethal Means (CALM), to train healthcare providers on how to counsel patients at risk of suicide on their access to lethal means, such as firearms.^10^,^11^ This training module also discusses strategies for safe storage of lethal means during times of suicidal crisis. In response to our findings, we have used these resources to implement a bedside CALM quality improvement initiative in our academic center ED for suicidal patients and their family members.

## Figures and Tables

**Figure:**
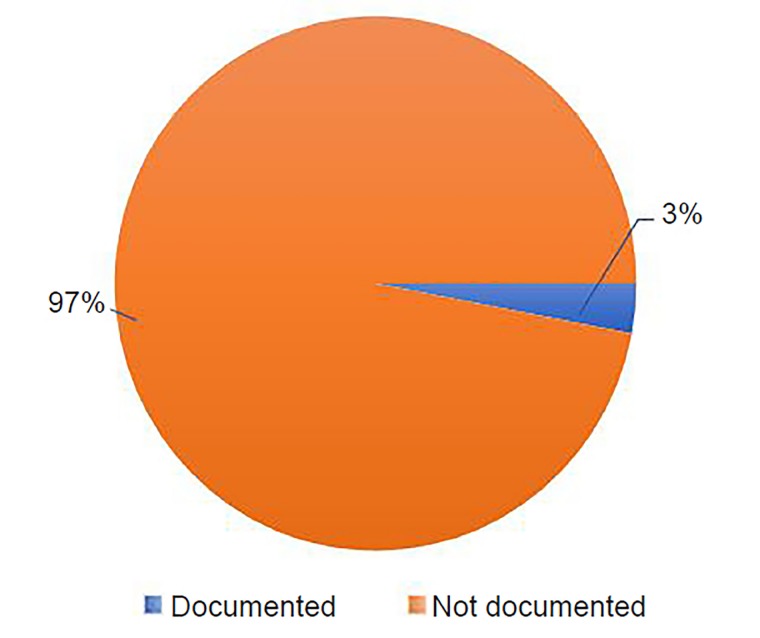
Emergency physician documentation of firearm access in 100 patients who presented to the emergency department for suicidal ideation between July 1–July 31, 2014.

**Table:** Demographics and characteristics of 100 patients who presented to the emergency department with suicidal ideation between July 1–July 31, 2014.

Age, number	
Age, median (IQR)	38 (26 – 47)
Age range	19 – 68
Sex, number	
Male	64
Female	36
Race, number	
African American	43
Caucasian	53
Other	4
Marital status, number	
Married	13
Partner	12
Single	54
Unknown	21
Psychiatric diagnoses, numer	
Anxiety	13
Bipolar	29
Depression	48
None	9
Psychosis, not otherwise specified	7
Schizophrenia	21
Other*	39
Disposition, number	
Admit	32
Discharge	32
Transfer	36

* Includes personality disorders, attention-deficit/hyperactivity disorder, and substance-induced mood disorders.

*IQR*, interquartile range.
